# Extrahepatic cytochrome P450 epoxygenases: pathophysiology and clinical significance in human gastrointestinal cancers

**DOI:** 10.18632/oncotarget.27893

**Published:** 2021-02-16

**Authors:** Nataliya Pidkovka, Olena Rachkevych, Abbes Belkhiri

**Affiliations:** ^1^Department of Health Science, South College, Nashville, TN, USA; ^2^Department of Obstetrics and Gynecology, Danylo Halytsky Lviv National Medical University, Lviv, Ukraine; ^3^Department of Surgery, Vanderbilt University Medical Center, Nashville, TN, USA; ^4^Vanderbilt-Ingram Cancer Center, Vanderbilt University Medical Center, Nashville, TN, USA

**Keywords:** cytochrome P450 epoxygenases, extrahepatic CYPs, gastrointestinal cancers, epoxyeicosatrienoic acids, arachidonic acid

## Abstract

Cytochrome P450 (CYP) epoxygenases, a multi-gene superfamily of heme-containing enzymes, are commonly known to metabolize endogenous arachidonic acid (AA) to epoxyeicosatrienoic acids (EETs). The role of CYPs is mostly studied in liver drugs metabolism, cardiac pathophysiology, and hypertension fields. Particularly, the biological functions of these enzymes have increasingly attracted a growing interest in cancer biology. Most published studies on CYPs in cancer have been limited to their role as drug metabolizing systems. The activity of these enzymes may affect drug pharmacokinetics and bioavailability as well as exogenous compounds turnover. Some CYP isoforms are selectively highly expressed in tumors, suggesting a potential mechanistic role in promoting resistance to chemotherapy. Majority of drugs elicit their effects in extrahepatic tissues whereby their metabolism can significantly determine treatment outcome. Nonetheless, the role of extrahepatic CYPs is not fully understood and targeting these enzymes as effective anti-cancer therapies are yet to be developed. This review article summarizes an up-to-date body of information from published studies on CYP enzymes expression levels and pathophysiological functions in human normal and malignant gastrointestinal (GI) tract tissues. Specifically, we reviewed and discussed the current research initiatives by emphasizing on the clinical significance and the pathological implication of CYPs in GI malignancies of esophagus, stomach, and colon.

## INTRODUCTION

The increasing incidence of gastrointestinal (GI) malignancies may be linked to a Westernization of lifestyle and risk behavior [1–3]. According to the World Health Organization estimate for the last few years, cancers of different parts of GI tract are considered among 15 most common malignancies with the highest rates of mortality [4–6]. Cytochrome P450 (CYP) epoxygenases include a superfamily of enzymes generally expressed in the liver, kidney, and the cardiovascular system [7]. Up to 18 mammalian CYP gene families and 44 subfamilies that include 57 human CYP genes have been identified [8]. CYP2, CYP3, and CYP4 are the largest human gene families [9]. These enzymes catalyze reactions involved in the metabolism of xenobiotics and lipids synthesis such as of cholesterol, bile acids, fatty acids and steroids [10]. Particularly, endogenous arachidonic acid (AA) as a substrate [11] is converted by CYP ω-hydroxylases to hydroxyeicosatetraenoic acids (HETEs) and by CYP epoxygenases to 5,6-, 8,9-, 11,12-, and 14,15 epoxyeicosatrienoic acids (EETs) [12]. EETs are potent endogenous vasodilators [13, 14], inhibitors of vascular inflammation [15], and contribute to blood pressure homeostasis through the regulation of Na^+^ transport in kidney epithelium [16, 17]. The role of CYPs and their metabolites in oncogenesis, angiogenesis, and metastasis is a popular field of research in eicosanoid biology [18].

CYPs highly expressed in the liver [19] play an important role in oxidative metabolism of xenobiotics and clearance of toxic drugs [20]. However, extrahepatic CYP enzymes and their biologically active lipid products might exhibit different tissue-specific functions. For instance, CYP-mediated metabolism in the gut wall affects the bioavailability of oral drugs [21, 22]. Orally administered xenobiotics undergo the bioactivation processes in target tissues following further detoxification through the digestive system associated CYPs [7]. While metabolism of xenobiotics commonly leads to detoxification of exogenous compounds, the CYP-mediated reactions can also produce toxic metabolites that increase risks of pathologies, including development of cancers and birth defects [10]. Therefore, alteration in CYPs function within alimentary canal could contribute to GI carcinogenesis and affect the response of tumors to chemotherapy.

### Expression of CYP epoxygenases in human GI tract

The differential expression of CYPs in different parts of alimentary canal suggests different biological functions of these enzymes and their products outside of the liver [23]. In fact, studies of CYPs expression, localization, and function in human esophagus highlight the capacity of these enzymes to activate DNA-reactive carcinogens. For instance, the protein expression of CYP1A, CYP2A, CYP2E1, CYP3A, and CYP4A enzymes has been shown in 25 normal tissue specimens of human esophageal mucosa [24]. Early immunohistochemical evidence of CYPs expression in intestinal mucosa suggested that human intestinal wall, in addition to its absorptive function, might metabolize exogenous substances [25]. Therefore, members of the CYP1A, CYP2C, CYP2D, CYP2E, and CYP3A subfamilies have been shown to be constitutively expressed in the human intestinal mucosa [26, 27]. The highest CYPs expression was detected in the duodenum and it progressively decreased distally to the ileum [28]. Interestingly, most highly expressed CYPs were detected from mid-villus to villous tip, with little expression in the crypts of Lieberkühn [26]. However, crypt-specific expression of CYP2W1 was demonstrated by immunohistochemical analysis of human fetal colon [29]. Notably, in the small intestine, CYP3A has been found as the most abundant isoform with 50–82% expression of the total intestinal CYPs [30].

Studies of RNA preparations from the human and rat intestine revealed the mRNA expression of CYP2J2 and its corresponding rat homologue CYP2J3 mostly in the small intestine and colon, while CYP2J proteins were expressed throughout the entire GI tract [31]. Particularly, high levels of CYP2J2 protein were detected in nerve cells of autonomic ganglia, epithelial cells, intestinal smooth muscle cells, and vascular endothelium [31]. Furthermore, NADPH-dependent AA conversion to EETs was demonstrated in microsomal fractions from human jejunum, and gas chromatography/mass spectrometry analysis confirmed the presence of EETs. The authors suggested that CYP2J associated products may be involved in the release of intestinal neuropeptides, regulation of intestinal motility, and transport of intestinal electrolytes [31].

Rylander and co-authors characterized CYP2S1 isoform, which is selectively expressed in the intestine, demonstrated its importance for extrahepatic xenobiotic metabolism [32]. Notably, using subcellular fractionation and immunostaining methods, CYP2S1 protein expression was localized in the endoplasmic reticulum [32]. Expression of several CYPs is frequently induced by accumulation of a specific substrate [33]. Tissue specific CYPs produce different ratios of EET regioisomers [34], thereby alterations in CYPs activity could reduce the availability of AA for other metabolic pathways. This affects the synthesis of other bioactive metabolites such as proinflammatory prostaglandins. Particularly, the changes in AA metabolism through cyclooxygenase (COX) and lipoxygenase (LOX) pathways is a common feature of numerous malignancies and has been shown to play key roles in cancer progression [35]. The synthesis of AA-derived biologically active lipid metabolites can take place in the tumor and inflamed stromal tissues [36]. A summary of CYP enzymes expression in normal GI tissues is shown in Table 1.

**Table 1:** Overview of CYP protein expression in normal GI tract tissues

GI organ/tissue localization	CYP isoforms	References
Normal tissues		
Esophagus	Mucosa: CYP1A, CYP2A, CYP2E1, CYP3A, CYP4A	[24]
	CYP2J2	
	C1A1, 1A2, 2A, 2E1, 2J2, 3A5	[31]
Stomach	CYP2J2	[31]
	CYP2S1	[32]
Intestine	Mucosa: CYP1A, CYP2C, CYP2D, CYP2E, CYP3A	[26, 49] [30]
	Enterocytes: CYP 3A4. CYP3A5, CYP2J2, CYP 2C9, CYP2C19, CYP2D6	
	Autonomic ganglion cells, epithelial cells, smooth muscle cells, and vascular endothelium: CYP2J2	[31] [29, 32]
	Fetal colon: CYP2W1, CYP2S1	

### Role of CYP epoxygenases in GI cancers

Multiple factors, including diet, infectious agents, environmental toxins and oral drugs have been associated with GI carcinogenesis [37]. Because CYP pathways mediate the effect of exogenous factors, studying the expression patterns and the activity changes of these enzymes in GI cancer tissues could lead to the development of new clinical and therapeutic approaches based on extrahepatic CYPs expression and activity in various GI cancers.

### Esophageal cancer

Esophageal cancer, which consists of two histological subtypes, esophageal squamous cell carcinoma (ESCC) and esophageal adenocarcinoma (EAC), represents the eighth most frequently occurring malignancy and the sixth most common cause of cancer-related death worldwide [6]. The known risk factors of ESCC include cigarette smoking, alcohol beverage consumption, and low intake of fruits and vegetables [38]. Chronic gastroesophageal reflux disease (GERD) and precancerous Barrett’s esophagus are major risk factors of EAC [39]. Disparity between phase I drug metabolism mediated by CYPs and phase II detoxification by other enzymes such as glutathione-S-transferases (GST) has been suggested as a contributing factor to pathogenesis of these cancers [40]. In fact, early immunohistochemical studies of esophageal cancer and non-neoplastic esophageal tissue samples revealed the expression of CYP1A, CYP2C, CYP3A and the functionally associated enzymes Epoxide Hydrolase (sEH) and GST in 60% of studied cancer samples [41]. Western blot analysis showed that CYP3A4, CYP3A5, and CYP2C8 protein levels in ESCC patients were significantly higher than in control group of healthy patients. Conversely, CYP2E1 protein level was significantly lower in ESCC patients than in healthy control group [42].

Surprisingly, the analysis of CYP1A, CYP2A, CYP2E1, CYP3A, and CYP4A protein levels in 25 non-neoplastic surgical tissue specimens of human esophageal mucosa did not reveal any significant associations with the patients’ medical history data and known esophageal cancer risk factors, such as tobacco smoke and GERD [24]. These unexpected results were explained by the small number of studied samples that impeded the authors from making any conclusions regarding the connection between CYPs expression and esophageal cancer. Studies of CYPs expression in esophagectomy specimens demonstrated the expression of CYP1A2, CYP3A4, CYP2E1, and CYP2C9/10 proteins in esophageal squamous mucosa and in the basal glandular actively proliferating areas of Barrett’s esophagus [43]. Another study showed that mRNA levels of CYP3A4 and CYP2C were significantly lower in malignant tissue than in normal tissue in ESCC patients [42].

CYP2J2, a well-characterized epoxygenase, is highly expressed in the cardiac tissues and vascular endothelium [15, 44]. In addition to its cardioprotective functions, the implication of CYP2J2 in carcinogenesis has been investigated intensively. Notably, abundant mRNA and protein expression of CYP2J subfamily has been demonstrated through the entire human and rat GI tract from esophagus to colon [31]. Moreover, high and selective CYP2J2 expression was demonstrated in various human tumor tissue samples and cancer cell lines [3, 45]. In fact, Jiang and co-authors [3] characterized CYP2J2 expression in tissue samples obtained from 130 patients with different types of cancer. In 77% of the patients, CYP2J2 mRNA and protein levels were markedly higher in tumors than surrounding noncancer tissues. The CYP2J2 expression was observed in most samples from all tumor types, including ESSC (20 of 31) and EAC (4 of 4) [3]. The data demonstrated that CYP2J2 plays an important role in the pathogenesis and progression of several types of human cancers [3]. Notably, CYP2J2 has been associated with inflammation and the pathogenesis of Crohn’s disease [46]. Interestingly, inhibition of CYP2J2 with terfenadine-related compounds decreased EET production and suppressed growth and proliferation in human tongue carcinoma cells and in murine xenograft models [45]. Together, these findings suggest that CYP2J2 might contribute to neoplastic pathogenesis of GI epithelium. Alterations of CYP expression in patients with ESCC support a potential role of these enzymes in the pathogenesis of esophageal malignancies. Additionally, the known ability of CYP system to activate carcinogens could lead to oncogenic transformation in metaplastic esophagus.

### Gastric cancer

Gastric cancer is one of the most frequent malignancies in the world and the third leading cause of cancer mortality [47]. *Helicobacter pylori* infection in combination with genetic polymorphisms associated with a predisposition to cancer development are risk factors for gastric carcinogenesis [48]. Based on early histochemical data, protein expression of CYP1A and CYP3A was detected in 51% and 28% of studied gastric cancer cases, respectively, and undetected in normal stomach tissues [49]. Additionally, the increased expression of CYP2J2 relative to adjacent normal tissue was shown in 5 out of 5 studied human gastric cancer samples [3]. These data support the CYP2J2 expression trend observed in other cancerous tissue samples [3]. Interestingly, a new report showed that elevated expression of CYP3A4 could be associated with the progression of chronic atrophic gastritis to gastric cancer and might predict poor prognosis [50]. A recent study has shed some light on the role of CYP2E1 in the development and progression of gastric adenocarcinoma [51]. Overexpression of CYP2E1 in gastric cancer cells enhanced proliferation, invasion, and survival. Mechanistic investigations showed that CYP2E1 overexpression upregulated the oncogenic PI3K-AKT-mTOR signaling pathway in gastric cancer cells [51]. Notably, little research on CYPs in the stomach was done because CYPs are generally expressed less in the normal gastric mucosa than in the other parts of the GI tract. The fact that gastric epithelium has rather a secretory than absorptive function in addition to the mucous barriers suggests that the stomach may be protected from chemical agents [52]. Therefore, the role of CYPs in driving gastric carcinogenesis remains largely unknown. Additional studies will be required to gain more information on the potential implication of CYPs in gastric cancer.

### Colon cancer

Colon cancer is the third occurring malignancy and the second leading cause of cancer mortality in the United States [53]. Hereditary syndromes, such as familial adenomatous polyposis and Lynch Syndrome, and inflammatory bowel disease are major predisposition risks for the development of colon cancer [54]. Tumor-specific expression of CYP2W1 was detected in approximately 30% of higher-grade colon cancers, while the expression of this enzyme was insignificant in normal colon tissues [55–57]. Interestingly, mouse and human developmental studies showed that CYP2W1 is expressed in the small intestine and colon tissues in the early stages of embryonic development and silenced shortly after birth [29]. Postnatal silencing of both murine and human *CYP2W1* gene was associated with increased methylation of CpG-rich promoter regions [58]. CYP2W1 expression can be induced by the treatment with the antitumor agent imatinib, linoleic acid and its derivatives in the colon adenocarcinoma cell line HCC2998 [29]. Although activation of CYP2W1 by demethylation in colorectal cancer (CRC) has been confirmed [58], the precise mechanisms of epigenetic modifications of *CYP2W1* gene remain unclear. The positive correlation of the increasing CYP2W1 expression with tumor progression and metastasis in CRC [59–61] could be used as a diagnostic tool. Larger scale clinical studies will be required to validate the potential application of CYP2W1 as a prognostic cancer biomarker.

A recent study based on a metabolomics approach demonstrated that epoxygenated fatty acids, which are eicosanoid metabolites produced by CYP epoxygenases, were elevated in the plasma and colon of azoxymethane (AOM)/dextran sodium sulfate (DSS)-induced colon cancer mouse model [62]. Genetic knockdown or pharmacologic inhibition of CYPs decreased AOM/DSS-induced colon tumorigenesis in mice. Unlike other eicosanoid metabolites, treatment with 12,13-epoxyoctadecenoic acid (EpOME) increased AOM/DSS-induced colon tumorigenesis *in vivo*. These findings demonstrate that the previously understudied CYP epoxygenases and their lipid metabolites contribute to colon tumorigenesis [62]. CYP enzymes expression in neoplastic GI tissues is summarized in Table 2.

**Table 2:** Overview of CYP protein expression in neoplastic GI tissues

GI organ/tissue localization	CYP isoforms	References
Neoplastic tissues		
Barrett’s esophagus/esophageal squamous mucosa	CYP1A2, CYP3A4, CYP2E1, CYP2C9/10	[43]
ESCC	CYP1A↑, CYP2C↑, CYP3A↑	[41]
	CYP3A4, CYP3A5, CYP2C8, CYP2E1	[42]
	CYP2J2↑	[3]
EAC	CYP2J2↑	[3]
Gastric cancer	CYP1A, CYP3A	[49]
	CYP2J2↑	[3]
Colon adenocarcinoma	CYP2J2↑	[3]
	CYP2W1↑	[55, 59]

### CYP epoxygenases in the activation of pro-carcinogens

The GI tract is exposed to various exogenous compounds, including pro-carcinogens and orally consumed drugs. CYPs expressed in GI tissues might be involved in the metabolic activation of potential carcinogens [63]. Extrahepatic tissues play a key role in the CYP-mediated metabolism of xenobiotic compounds affecting the susceptibility of certain organs to neoplastic transformation [64]. It is established that chronic exposure to cigarette smoke and chewing tobacco have been associated with the development of esophageal cancer [65, 66]. The expression of xenobiotic-metabolizing CYPs in the esophagus may determine the susceptibility of this organ to the carcinogenic effect of tobacco-derived nitrosamines [67]. A study of esophageal microsomal samples from patients in the United States and Henan Province (China), a high-risk area for esophageal cancer, demonstrated that CYP3A4 and CYP2E1 are involved in the activation of tobacco carcinogens N’-nitrosonornicotine and N-nitrosodimethylamine, respectively, in the human esophagus [66]. The activities of xenobiotic-metabolizing enzymes were decreased by 30–50% in the squamous cell carcinomas as compared to their corresponding non-cancerous mucosa [66]. Another potent esophageal carcinogen, N-methyl-N-pentyl-nitrosamine, was metabolized by CYPs in microsomal fractions of human and rat esophagus [68].

### CYP epoxygenases as cancer drugs metabolizing systems

Xenobiotic-metabolizing CYPs in GI tissues are involved in the first-pass clearance and could contribute to the activation of anticancer drugs [7, 63]. The limitation to the current knowledge is that CYP-mediated metabolism has been investigated mostly in the liver and the drug-metabolizing function of GI CYPs remains incomplete. The anticancer drugs tamoxifen and cyclophosphamide have been shown to be metabolized by CYP2D6, CYP2C19 and CYP2B6 [63]. Notably, the CYP3A subfamily enzymes play a key role in the metabolism of approximately 30% of all clinically used drugs [69, 70]. Anticancer drugs metabolized by CYP3A include paclitaxel, ifosfamide, tamoxifen [69, 71, 72], and irinotecan [73]. A potential substrate-overlap between CYP3A4 and the multidrug resistance protein 1 (Mdr1) has been proposed [30, 74]. While selective expression of CYPs in GI tumors suggests a mechanism for drug resistance, both CYP- and Mdr1-mediated pathways may synergistically contribute to the metabolism and detoxification of oral drugs.

Gene-Directed Enzyme Prodrug Therapy (GDEPT) utilizing drug metabolizing CYPs that activate bio-reductive cytotoxins is a novel approach in increasing the efficacy of targeted therapy of drug-resistant hypoxic tumors. Hypoxia is a restricting factor in the clinical outcome of conventional cancer therapies, and promotes the malignant tumor progression [75]. The anti-cancer prodrug AQ4N [1,4-bis [2-(dimethylamino-*N*-oxide) ethyl] amino 5, 8-di-hydroxyanthracene-9, 10-dione] (banoxantrone) is converted by CYPs to the cytotoxin AQ4 in the hypoxic tumor microenvironment. AQ4N does not bind to DNA, while its derivative AQ4 has a high DNA affinity and is a potent topoisomerase II inhibitor [76], preventing tumor cells from proliferating [77, 78]. Targeting topoisomerase II, a key player in cell cycle regulation, may also sensitize tumors to radiotherapy. AQ4N, as a potential hypoxia-activated cancer chemotherapy drug, underwent clinical trials (NCT00394628) [79]. It has been shown that intra-tumoral injection of CYP3A4, CYP2B6 [77] and CYP1A1 [80] gene constructs in combination with AQ4N and radiation suppresses the growth of tumors in RIF-1 sarcoma mouse model. To our knowledge, there are no known GDEPT studies on GI cancer models. Indeed, because of their bio-metabolic characteristics, GI tumors may be a good candidate for radio-sensitization by exogenous CYPs-mediated drugs activation in a hypoxic tumor microenvironment.

Tumor-specific expression of CYP2W1 and its ability to activate multiple prodrugs to cytotoxic metabolites in mouse xenograft models of colorectal carcinoma (CRC) suggest that this enzyme may be an important target for CRC treatment [57, 60]. An approach was proposed to use CYP2W1 as a new tumor-associated antigen for cancer immunotherapy [57]. Notably, the imatinib-induced expression of tumor CYP2W1 followed by activation of duocarmycin prodrugs was suggested as an adjuvant therapy of CRC [57]. Additionally, CYP2W1 was reported to metabolize high affinity exogenous indolines, especially chloromethylindolines, into cytotoxic metabolites that inhibit growth of human colon tumors in a mouse xenograft model [60, 61]. The CRC specific expression of CYP2W1 and its effective activation of prodrugs makes it a valuable target for novel cancer therapeutics. Although CYP2W1 substrates comprise various endogenous compounds, including arachidonic acid, retinoic acid, and lysophospholipids [60, 61], additional substrates for this enzyme need to be investigated and further studies are required to verify if CYP2W1 is a specific drug target in CRC treatment.

An increasing body of evidence indicates that many medications, including those used in cancer treatment, are substrates for CYP2J2 [81]. CYP2J2 and CYP3A4 play a key role in the metabolism of cancer drugs known to cause cardiotoxicity [82]. The most studied case is the chronic and irreversible dose-dependent toxicity induced by Doxorubicin (DOX), an anthracycline used for the treatment of solid tumors and hematologic carcinomas. Frequent adverse side effects related to DOX include acute cardiomyopathy, chronic heart failure, and arrhythmias [82, 83]. Production of reactive oxygen species has been shown as the underlying mechanism of DOX cardiotoxicity [84]. Furthermore, DOX and its metabolite 7-deoxydoxorubicin aglycone (7-de-aDOX) have been shown to inhibit CYP2J2-mediated synthesis of EETs through binding to the active site of the enzyme [85]. It is highly possible that the metabolic activity of GI tract associated CYPs could also contribute to the cardiotoxicity of anti-cancer drugs. More studies on how the expression and activity of CYP2J and CYP4A in the digestive system affect drug concentration, production and elimination of toxic metabolites can help in the development of new effective cancer drugs. A summary of GI associated CYP enzymes that are important for the cancer drug metabolism is shown in Table 3.

**Table 3:** CYPs of clinical importance for cancer drug metabolism and clearance expressed in the GI tract

CYP isoforms	Substrates	References
CYP3A, CYP3A4	Taxol, Ifosfamide, Tamoxifen	[71]
	Irinotecan	[72]
	Docetaxel	[73]
	Cyclophosphamide	[100]
CYP2B6	Tamoxifen	[71, 100, 101]
	Cyclophosphamide	
CYP2C19	Tamoxifen	[63, 100]
	Cyclophosphamide	
CYP2C9	Cyclophosphamide	[100]
CYP2D6	Tamoxifen	[70, 72, 100]
	Cyclophosphamide	
CYP2J2	Tamoxifen	[102]
	Doxorubicin	[84, 85]
CYP2S1	1,4-bis [2-(dimethylamino-N-oxide) ethyl] amino 5, 8-di-hydroxyanthracene-9, 10-dione (AQ4N)	[103–105]
	N-hydroxylamine drug 2-(4-amino-3-methylphenyl)-5-fluorobenzothiazole	
CYP2W1	Chloromethylindolines	[59, 60]

### CYP polymorphisms and gastric cancer risk

The drug-metabolizing function of CYPs can be affected by genetic polymorphism of these enzymes. Adverse side effects and cancer therapy failure are associated with individual CYPs variability in drug pharmacokinetics and response [69, 86]. Most of human CYPs implicated in xenobiotics turnover belong to CYP1, CYP2, CYP3, and CYP4 families [10]. Multiple allelic variants within each of these gene families generate a high level of pharmacogenetic heterogeneity. Although several studies have established a link between genetic polymorphisms of CYPs and various pathologies, it remains unclear whether genetic polymorphisms of CYPs are associated with increased risks of GI cancers.

While multiple studies have shown that CYP1A1 genetic polymorphisms (CYP1A1 Msp I and CYP1A1 Ile/Val) could be risk factors for esophageal, gastric, and colorectal cancers [87], the current data remain controversial. Notably, a meta-analysis of the published data of CYP1A1 and CYP1A2 polymorphisms in different ethnicities revealed possible associations between CYP1A1 MspI and CYP1A2*1F polymorphisms and gastric cancer, and no significant associations between CYP1A1 Ile462Val polymorphism and gastric cancer [88]. Conversely, a meta-analysis of available clinical data in the Chinese population performed by Liu and colleagues demonstrated that CYP1A1 Ile/Val genetic polymorphisms, but not CYP1A1 MspI polymorphisms, are associated with an increased GI cancers risk [87]. Particularly, the study of single nucleotide polymorphisms (SNPs) of CYP1A1 (rs4646421, rs4646422, and rs1048943), GSTM1, and GSTT1, the key enzymes in the carcinogen metabolizing pathway, revealed that CYP1A1 (rs4646422) polymorphism could be implicated in gastric carcinogenesis in the Japanese population [89]. Interindividual variation in CYP2J2 expression has been assessed in relation to genetic polymorphism. Notably, 10 distinct star alleles have been identified [69]. The most common *CYP2J2* allele variant with functional relevance is CYP2J2*7, which arises at frequencies of 2–17% in various populations. The key SNP, rs890293, is in the proximal promoter at (−76 G>T) and disrupts one of the SP1 binding sites, which results in 50% decrease of promoter activity as compared to the wild-type promoter [90].

Pharmacogenetic studies in patients with gastric ulcers under *H. pylori* eradication therapy demonstrated the effect of *CYP2C19* polymorphism on pharmacokinetics of proton pump inhibitors (PPI), omeprazole, lansoprazole, pantoprazole, and to a lesser extent, rabeprazole [91, 92]. Therefore, genotypes of *CYP2C19* were classified as rapid metabolizers (RM: *1/*1), intermediate metabolizers (IM: *1/*X), and poor metabolizers (PM: *X/*X). *1 and *X represent the wild-type and mutant allele, respectively [93]. The pharmacokinetics and pharmacodynamics of PPIs varied among these three *CYP2C19* genotype groups. The lowest plasma PPI levels and intragastric pH following PPI treatment were the lowest in the RM group, intermediate in the IM group, and the highest in the PM group [93]. Several pharmacogenomic studies that include patients of different ethnicities demonstrated that PPI induce the increase of intragastric pH, promote ulcer healing, and improve efficacy of the antibiotics and overall treatment outcome according to the *CYP2C19* polymorphism [93–96]. Since PPIs are commonly used in the treatment of reflux esophagitis, gastroesophageal reflux disease (GERD), Zollinger–Ellison syndrome, non-ulcer dyspepsia, and NSAID-related damage, the healing process in the therapy of these diseases was predictably affected by *CYP2C19* genotype [91, 93, 96, 97]. Surprisingly, esomeprazole-induced healing of GERD was not associated with the *CYP2C19* polymorphism and was explained by the CYP3A4 metabolic activity [98]. Therefore, personalized *H. pylori* eradication protocols that include inhibition of excessive acid secretion and antimicrobials can achieve higher eradication rates, improve healing process, and prevent neoplastic transformation of gastric epithelium.

Knowledge of the intrinsic and extrinsic factors that regulate expression and function of the CYP enzymes is a requirement for predicting variable pharmacokinetics and drug treatment response. While monogenic polymorphisms explain the variability for only few enzymes, most enzymes are controlled by several factors that include additional polymorphisms in regulatory genes and factors such as sex, age, disease, and hormones [69].

## CONCLUSIONS AND PERSPECTIVES

**Figure 1:**
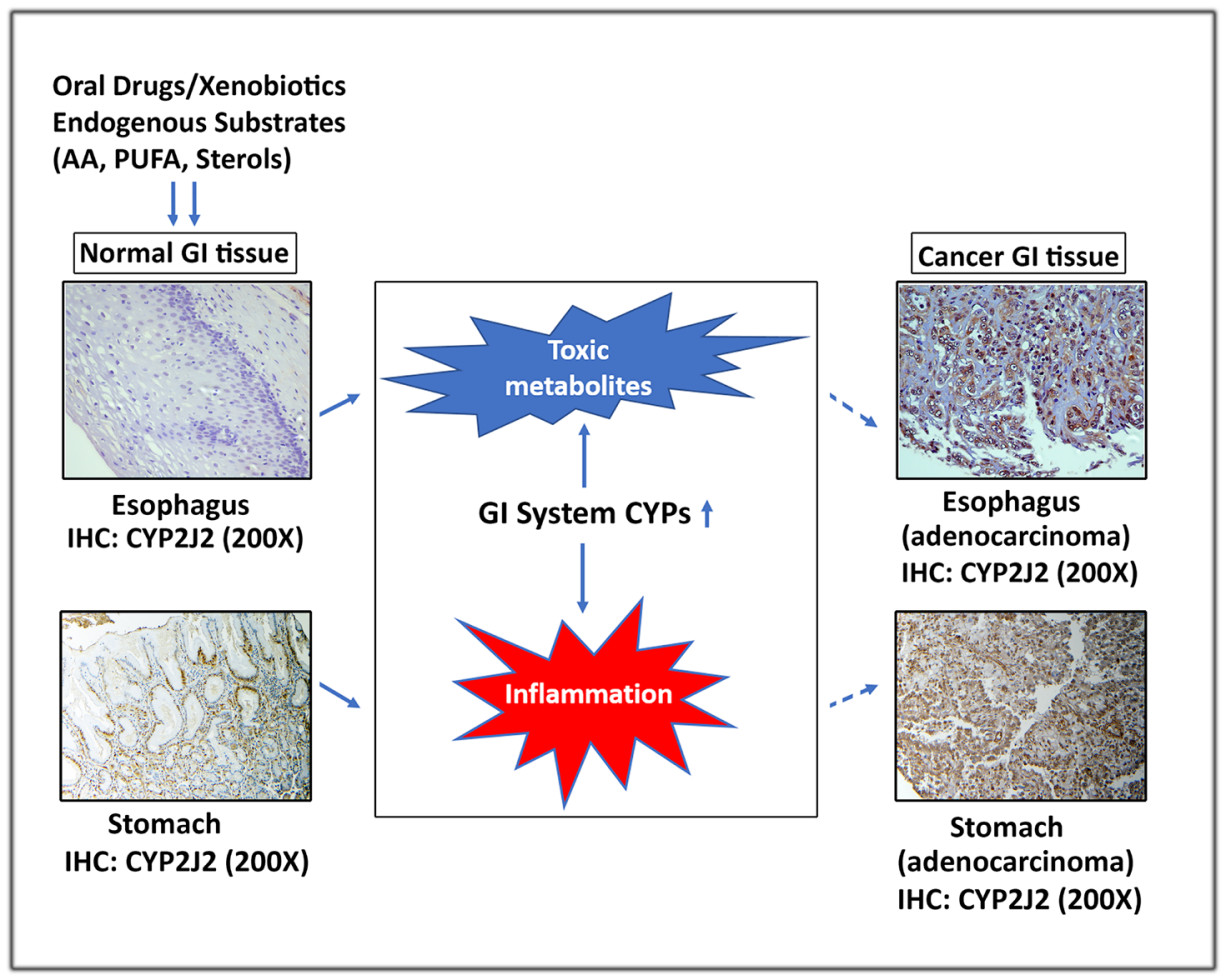
A schematic representation depicting the role of extrahepatic GI system CYPs in carcinogenesis. Exposure of normal GI tissues to xenobiotics and endogenous substrates (AA, PUFA & sterols) alters expression of CYPs, leading to production of toxic and proinflammatory metabolites that contribute to development of GI cancers. For illustration, protein of CYP2J2 was evaluated by IHC in normal and cancer GI tissues.

There is a growing body of evidence that changes in the CYPs expression and enzymatic activity may play a major role in the GI cancer pathogenesis and progression. The expression of CYPs varies throughout the different parts of alimentary canal and this pattern is altered in cancerous tissues. Xenobiotics following absorption through intestinal wall and their metabolism by intestinal CYPs pass to liver through the portal vein [55, 99]. Hepatic CYPs in a coordinated fashion perform the final stages of drug metabolism to maintain the capacity of the digestive organs for first-pass clearance of orally administered drugs [99]. Some studies have recently implicated the CYP metabolites in inflammation and tumorigenesis [62]. The published data strongly suggest that exposure of GI tract-associated tissues to endogenous and exogenous substrates alters expression of CYPs, leading to production of proinflammatory metabolites that play a key role in tumorigenesis (Figure 1).

The available clinical data point to an important role for GI tract associated extrahepatic CYPs in cancer pathogenesis. Notably, increased expression of individual CYPs in GI cancer cells provides an opportunity for development of compounds that would be specifically metabolized. In addition, selective expression of CYPs in GI tumors strongly suggests a mechanism for drug resistance. Targeting CYP-mediated mechanisms of cancer drugs breakdown could help to address the major challenges in current chemotherapies, especially drug toxicity and resistance. The CYP2C19 genetic polymorphism has been shown that it could predict the clinical outcome of patients with gastric ulcers or GERD treated with PPIs [91, 93, 96, 97]. This pharmacogenetic approach could be applied to individual GI cancer patients to predict sensitivity or resistance to anti-cancer drugs. The development of this precision medicine strategy requires extensive genotyping of highly expressed CYPs in GI tumors and assessing the pharmacokinetics of various drugs and response to treatments. It is plausible that metabolizing drugs by specific CYP genotypes could lead to drug neutralization or generation of more toxic metabolites for tumors. Additionally, activation of bio-reductive cytotoxins through CYP gene-directed prodrug therapy is another potentially highly effective approach in the targeted treatment and radio-sensitization of drug-resistant hypoxic GI tumors. More studies investigating the mechanisms of CYPs expression and activity in the digestive tract system in relation to the drug-metabolizing functions will be required for the development of new and effective targeted cancer therapeutic approaches.

## Abbreviations

AA: Arachidonic acid
EAC: Esophageal adenocarcinoma
AOM: Azoxymethane
COX: Cyclooxygenase
CRC: Colorectal carcinoma
GI: Gastrointestinal
CYP: Cytochrome P450 epoxygenase
DOX: Doxorubicin
DSS: Dextran sodium sulfate
EET: Epoxyeicosatrienoic acid
ESCC: Esophageal squamous cell carcinoma
EpOME: Epoxyoctadecenoic acid
FAP: Familial adenomatous polyposis
GDEPT: Gene-directed enzyme prodrug therapy
GERD: Gastroesophageal reflux disease
GST: Glutathione S transferase
HETE: Hydroxyeicosatetraenoic acid
LOX: Lipoxygenase
PPI: Proton pump inhibitor
PUFA: Polyunsaturated fatty acid
ROS: Reactive oxygen species
sEH: Soluble epoxide hydrolase
